# Anti-hypertensive medication adherence in the REQUIRE trial: post-hoc exploratory evaluation

**DOI:** 10.1038/s41440-023-01333-8

**Published:** 2023-06-01

**Authors:** Kazuomi Kario, Hisashi Kai, Shinsuke Nanto, Hiroyoshi Yokoi

**Affiliations:** 1 Division of Cardiovascular Medicine, Department of Medicine, Jichi Medical University School of Medicine, Tochigi, Japan; 2 Department of Cardiology, Kurume University Medical Center, Fukuoka, Japan; 3 Department of Cardiovascular Medicine, Nishinomiya Municipal Central Hospital, Hyogo, Japan; 4 Cardiovascular Center, Fukuoka Sanno Hospital, Fukuoka, Japan

**Keywords:** Blood pressure, Hypertension, Medication adherence, Renal denervation

## Abstract

Maintaining medication adherence is important in treating hypertension, especially resistant hypertension (RH), and variable medication adherence can confound results in blood pressure trials. This post-hoc analysis evaluated adherence at baseline and 3 months using available urine samples from the REQUIRE trial, comparing 24-h ambulatory systolic blood pressure (ASBP) lowering effects of ultrasound renal denervation (uRDN) versus sham in RH. At baseline, 45% (26/58) patients showed poor adherence. Among patients with good baseline adherence, adherence was unchanged at 3 months, and uRDN patients had a decreased ASBP whereas sham patients did not. In poorly adherent patients, sham patients showed a trend towards increased adherence and a significant ASBP reduction, whereas uRDN patients did not change. Accordingly, adherence changes and the resultant ASBP reduction in poorly adherent sham patients may explain the lack of between-group difference seen in REQUIRE. Monitoring and maintaining medication adherence is important for future interventional studies in RH.

## Introduction

The sympathetic nervous system plays an important pathophysiological role in hypertension [1]. Renal sympathetic nerve denervation (RDN) is a promising non-pharmacological treatment for uncontrolled hypertension. Multiple basic science and clinical trials of RDN [2, 3] have evaluated its clinical application [4] and have accumulated clinical evidence supporting its use in treating hypertension and related comorbidities.

Recent meta-analyses have reported a significant blood pressure (BP)-lowering effect of RDN compared to a sham procedure [5, 6]. To date, four sham-controlled trials of the Paradise^TM^ Ultrasound RDN System (ReCor Medical, Inc., Palo Alto, CA, USA) have been conducted for patients with uncontrolled hypertension, including resistant hypertension (RH), which is defined as uncontrolled BP while on ≥3 antihypertensive medications including a diuretic. The RADIANCE-HTN SOLO, RADIANCE-HTN TRIO and RADIANCE II trials, which were conducted in the United States and Europe, reported that ultrasound RDN (uRDN) significantly reduced daytime and 24-h ambulatory systolic BP (ASBP) compared to the sham procedure [7–9]. In contrast, the REQUIRE trial conducted in Japan and South Korea demonstrated no significant difference in ASBP changes between uRDN- and sham-treated RH patients at 3 months [10]. In REQUIRE, the extent of uRDN-induced ASBP reduction was comparable to that in other uRDN trials, whereas the ASBP reduction in the sham group was unexpectedly larger [7–9]. In REQUIRE, there was a large, gradual BP decrease recorded over the first 3 months leading to an equalization of blood pressure between the uRDN and sham groups at 3 months. Although changes in concomitant medication adherence was a hypothesized contributor to the results, until now, we have not directly assessed this. In this post-hoc analysis from the REQUIRE trial, urine samples were tested for concomitantly-prescribed anti-hypertensive drugs or their metabolites in order to evaluate medication adherence.

## Methods

The details of the REQUIRE trial was described as previously reported [10] and this study was conducted in accordance with the Ethical Guidelines for Medical and Health Research Involving Human Subjects issued by the Ministry of Health, Labour, and Welfare. Acknowledgment for this post-hoc analysis was obtained from the primary investigators and study sites prior to analysis. The urine samples were stored at −20 °C avoiding light at LSI Medience Corporation (Tokyo, Japan). Only urine samples from the cohort of patients from Japan were evaluated, because samples from patients from South Korea had been discarded prior to this investigation. Only patients, whose urine samples were available at both baseline and 3 months post-procedure were included. The urine tests were qualitatively performed at the Department of Experimental and Clinical Toxicology at Saarland University (Homburg, Germany) using liquid chromatography/high-resolution mass spectrometry [11]. Medication adherence was defined as the ratio (%) of the number of drugs detected to the number of drugs prescribed at the time of urine sample collection. Adherence rates were classified into four categories: full adherence (100%), greater partial adherence (75–99%), lesser partial adherence (1–74%), and non-adherence (0%). The proportion of patients classified into each category was summarized. Adherence rates ≥75% at baseline were designated as good adherence and <75% as poor adherence. Data were presented as the mean ± standard deviation and the proportion of patients.

## Results and discussion

Of the 136 RH patients enrolled in the REQUIRE trial, 58 were assessed in this analysis. Baseline characteristics of the cohort with available urine data were similar to those of the entire REQUIRE cohort (Supplementary Table 1).

**Fig. 1:**
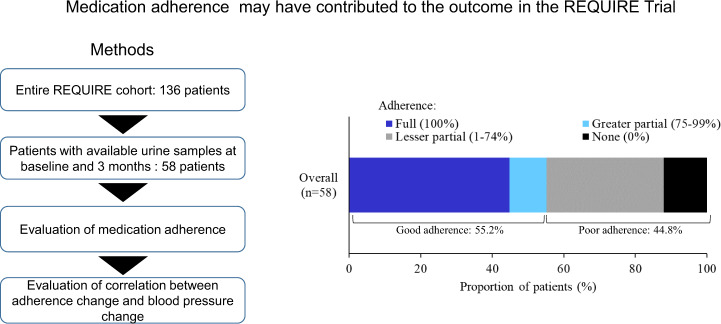
Graphical abstract: Medication adherence at baseline. 55.2% of patients with resistant hypertension showed good adherence, whereas 44.8% showed poor adherence

At baseline, 55.2% showed good medication adherence (full: 26 patients, greater partial: 6 patients) and 44.8% had poor adherence (lesser partial: 19 patients, none: 7 patients) (Fig. 1). To the best of our knowledge, this is the first study to conduct a direct evaluation of medication adherence in Japanese patients with RH, though the number of patients studied was small. The proportion of patients with good adherence in our study was consistent with the results from DENERHTN [12] and the Peregrine studies [13], demonstrating low adherence rate of 40–50% for RH patients. Thus, the REQUIRE trial seems to have included many patients with pseudo-RH, or poor BP control due to poor medication adherence.

We evaluated differences in baseline characteristics between patients with good adherence and poor adherence. In the poor adherence group, age (51.0 ± 9.4 vs 58.5 ± 11.4, *p* = 0.010 by the unpaired *t*-test) at baseline was lower, and the number of prescribed antihypertensive drugs (3.9 ± 1.6 vs 3.1 ± 1.0, counted on the basis of formulation, *p* = 0.0214 by the unpaired *t*-test) and baseline ASBP (170.8 ± 10.7 mmHg vs 160.8 ± 14.9 mmHg, *p* = 0.006 by the unpaired *t*-test) were higher.

**Fig. 2:**
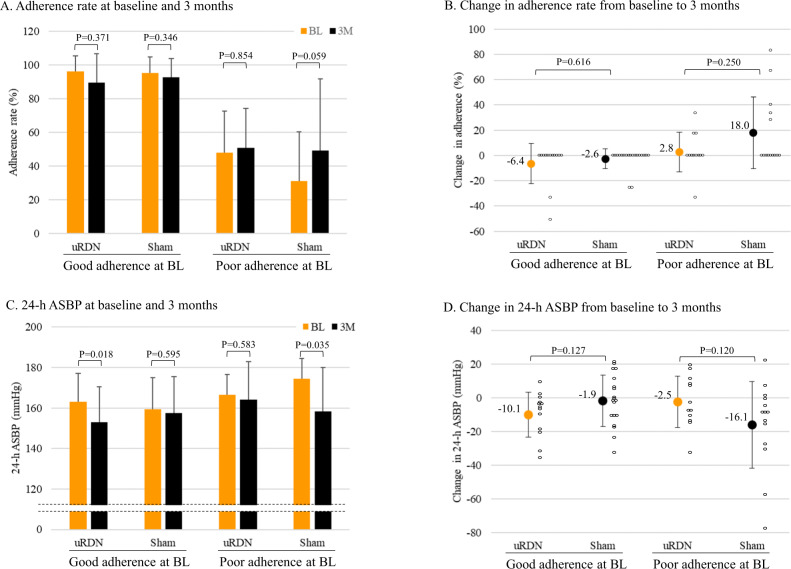
Change in medication adherence rate and 24-h ambulatory systolic blood pressure after ultrasound renal sympathetic nerve denervation or sham procedure. Actual value at baseline and 3 months for adherence (**A**) and its change from baseline (**B**). Actual value at baseline and 3 months for 24-h ambulatory systolic blood pressure (**C**) and its change from baseline (**D**). Circles indicate individual values. Data are expressed as the mean + or ± standard deviation. The Wilcoxon signed-rank test and the paired *t*-test were performed for comparisons of adherence rate and ASBP between baseline and 3 months post-procedure, respectively. The Mann–Whitney *U* test and the unpaired *t*-test were performed for comparisons of change in adherence rate and ASBP between two groups, respectively. Values are described in Supplementary Table 3

Next, we examined the effects of baseline medication adherence on the changes in adherence and ASBP at 3 months post-procedure. Figure 2A demonstrates that adherence rates did not change in patients with good adherence undergoing either uRDN or sham and poorly adherent patients undergoing uRDN. However, in poorly adherent patients undergoing sham procedure, medication adherence showed a trend towards improvement (*p* = 0.059). Fig. 2C shows that in patients with good adherence, uRDN decreased ASBP significantly (*p* = 0.018), whereas sham did not. In poorly adherent patients, ASBP did not change in the uRDN group, but decreased in the sham group (Fig. 2C, *p* = 0.035).

In addition, the reduction in ASBP following uRDN compared to sham was −10.1 ± 13.3 mmHg versus −1.9 ± 15.3 mmHg, respectively, in patients with good adherence at baseline (Fig. 2D and Supplementary Table 3), which was comparable to that reported in RADIANCE-HTN SOLO, RADIANCE-HTN TRIO and RADIANCE II trials demonstrating the BP-lowering effect of uRDN superior to sham-operated patients [7–9].

It was noteworthy that three (21%) of 14 sham-operated patients with poor adherence at baseline showed remarkable ASBP reductions greater than −30 mmHg (Fig. 2D). Such large BP reductions were likely due to improved medication adherence that was poor before the study, rather than to the effect of the therapeutic intervention. Overall, adherence was improved in five (36%) of 14 sham-operated patients with poor adherence at baseline (Fig. 2B).

Given the adherence change over time in some patients, an additional analysis was conducted to look at patients that had good adherence at both baseline and 3 months. A significant BP-lowering effect of uRDN was evident in patients who had good baseline medication adherence and maintained good adherence during the observation period (24-h ASBP, 165.2 ± 14.3 mmHg at baseline, 155.3 ± 18.1 mmHg at 3 months, *n* = 11, *p* = 0.042 by the paired *t*-test), whereas the sham did not show a significant decrease (159.3 ± 1.7 mmHg at baseline, 157.4 ± 18.2 mmHg at 3 months, *n* = 19, *p* = 0.595 by the paired *t*-test).

Although the number of patients in this analysis was a bit less than half of the entire REQUIRE cohort, the baseline patient characteristics were similar. These data suggest that the gradual and large BP reduction in the sham group is in part explained by improvement of medication adherence during the observation period. Taken together, it is possible that REQUIRE included many patients who originally had poor BP control due to poor medication adherence and the BP-lowering effect of uRDN was offset by improved medication adherence during the observation period in such patients, particularly in the sham group.

This study had limitations. First, the long-term stability of drugs in urine samples has not been confirmed. Second, a qualitative measurement was employed; therefore, information about the exact drug doses could not be obtained. Third, the pharmacokinetic characteristics of the drugs and renal and liver function of patients could have influenced the detection of the compound; and several drugs were not measured because they were not listed in the drug panels able to be measured (Supplementary Table 2). A sensitivity analysis including the 48 patients in whom all prescribed drugs or their metabolites were able to be measured confirmed the results described above (Supplementary Tables 4–6).

In conclusion, this REQUIRE post-hoc analysis showed that about 45% of Japanese RH patients had poor medication adherence. A significant BP-lowering effect of uRDN could be evident in patients having good baseline medication adherence that was maintained during the observation period. Monitoring and maintaining medication adherence is important for future intervention studies in RH.

## Supplementary information

Supplementary information
