# Supplementary material for: Anti-hypertensive medication adherence in the REQUIRE trial: post-hoc exploratory evaluation
Source: Hypertens Res. 2023 Jun 1;46(8):2044–7. doi: 10.1038/s41440-023-01333-8 (PMC10404509; doi:10.1038/s41440-023-01333-8)
Supplement: Supplementary file 1 — Supplementary information [file 41440_2023_1333_MOESM1_ESM.docx]

Supplementary information

Anti-hypertensive medication adherence in the REQUIRE trial: Post-hoc exploratory evaluation

Kazuomi Kario, Hisashi Kai, Shinsuke Nanto, Hiroyoshi Yokoi

Supplementary Table 1. Baseline patient characteristics

|  | Patients investigated for adherence (n = 58) | Entire REQUIRE cohort (n = 136) |
| --- | --- | --- |
| Age, years | 55.1 ± 11.1 | 53.1 ± 11.9 |
| Female, n (%) | 13 (22.4) | 35 (25.7) |
| Body mass index, kg/m^2^ | 28.8 ± 5.1 | 28.9 ± 5.0 |
| eGFR, mL/min per 1.73 m^2^ | 72.0 ± 16.9 | 71.9 ± 16.7 |
| 24-h systolic ambulatory blood pressure, mmHg | 165.3 ± 14.0 | 161.7 ± 13.2 |
| Number of antihypertensive drugs, n (%)  3  4  ≥ 5 | 30 (51.7)  15 (25.9)  13 (22.4) | 61 (44.9)  43 (31.6)  32 (23.5) |

Data are expressed as the mean ± standard deviation, or number of patients (%)

*eGFR* estimated glomerular filtration rate

Number of antihypertensive drugs were counted on the basis of active ingredient.

Supplementary Table 2. List of anti-hypertensive drugs unable to be measured in this study.

| Drug | Reason |
| --- | --- |
| Benidipine  Arotinolol  Guanabenz | Not detectable by liquid chromatography/high-resolution mass spectrometry |
| Azelnidipine  Cilnidipine | Not detectable due to instability to lights |

For the calculation of adherence rate, the listed drugs above were considered as not prescribed.

Supplementary Table 3. Change in medication adherence and 24-h systolic ambulatory blood pressure after ultrasound sympathetic nerve denervation and sham procedure

| Category | Group | Adherence, % | | 24-h ASBP, mmHg | |
| --- | --- | --- | --- | --- | --- |
|  |  | Baseline | 3 Months/  Change | Baseline | 3 Months/  Change |
| Good adherence | uRDN  (n = 13) | 96.2 ± 9.4  - | 89.7 ± 17.1  -6.4 ± 16.0 | 163.0 ± 14.1  - | 152.9 ± 17.7  -10.1 ± 13.3 |
|  | Sham  (n = 19) | 95.3 ± 9.5  - | 92.6 ± 11.2  -2.6 ± 7.9 | 159.3 ± 15.7  - | 157.4 ± 18.2  -1.9 ± 15.3 |
| Poor adherence | uRDN  (n = 12) | 48.1 ± 24.7  - | 50.8 ± 23.7  2.8 ± 15.6 | 166.6 ± 10.0  - | 164.1 ± 18.8  -2.5 ± 15.3 |
|  | Sham  (n = 14) | 31.3 ± 29.0  - | 49.3 ± 42.6  18.0 ± 28.3 | 174.4 ± 10.2  - | 158.2 ± 21.7  -16.1 ± 25.6 |

Data are expressed as the mean ± standard deviation

*24-h ASBP* 24-h systolic ambulatory blood pressure; *uRDN* ultrasound renal sympathetic nerve denervation

Supplementary Table 4. Baseline patient characteristics in the sensitivity analysis population in whom all prescribed drugs or their metabolites were measured.

|  | Patients investigated for adherence (n = 48) |
| --- | --- |
| Age, years | 55.9 ± 11.4 |
| Female, n (%) | 9 (18.8) |
| Body mass index, kg/m^2^ | 28.9 ± 5.3 |
| eGFR, mL/min per 1.73 m^2^ | 71.1 ± 16.4 |
| 24-h systolic ambulatory blood pressure, mmHg | 165.3 ± 14.8 |
| Number of antihypertensive drugs, n (%)  3  4  ≥ 5 | 26 (54.1)  13 (27.1)  9 (18.8) |

Data are expressed as the mean ± standard deviation, or number of patients (%)

*eGFR* estimated glomerular filtration rate

Supplementary Table 5. Medication adherence at baseline in the sensitivity analysis population

|  | Adherence |
| --- | --- |
| Overall (n=48) | Good adherence (≥75%): 28 (58.3)  Full (100%): 22 (45.8)  Greater partial (75-99%): 6 (12.5) |
|  | Poor adherence (<75%): 20 (41.7)  Lesser partial (1-74%): 16 (33.3)  Non (0%): 4 (8.3) |

Data are expressed as number of patients (%)

Supplementary Table 6. Change in medication adherence and 24-h systolic ambulatory blood pressure between baseline and 3 months post-procedure in the sensitivity analysis population

| Category | Group | Adherence/ Change from baseline, % | | 24-h ASBP/ Change from baseline, mmHg | |
| --- | --- | --- | --- | --- | --- |
|  |  | Baseline | 3 months | Baseline | 3 months |
| Good adherence | uRDN  (n = 9) | 94.4 ± 11.0  - | 94.4 ± 11.0  0.0 ± 0.0 | 166.3 ± 15.6  - | 157.2 ± 19.6  -9.1 ± 15.2 |
|  | Sham  (n = 19) | 95.3 ± 9.5  - | 92.6 ± 11.2  -2.6 ± 7.9 | 159.3 ± 15.7  - | 157.4 ± 18.2  -1.9 ± 15.3 |
| Poor adherence | uRDN  (n = 11) | 46.4 ± 25.2  - | 46.4 ± 18.9  0.0 ± 12.9 | 165.7 ± 10.0  - | 163.7 ± 19.7  -2.0 ± 15.9 |
|  | Sham  (n = 9) | 38.0 ± 29.2  - | 58.3 ± 40.8  20.4 ± 33.1 | 176.2 ± 11.8  - | 150.4 ± 21.5  -25.8 ± 26.2 |

Data are expressed as the mean ± standard deviation

*24-h ASBP* 24-h ambulatory systolic blood pressure; *uRDN* ultrasound renal sympathetic nerve denervation
